# Case Report: Transformation of Visual Snow Syndrome From Episodic to Chronic Associated With Acute Cerebellar Infarct

**DOI:** 10.3389/fneur.2022.811490

**Published:** 2022-02-15

**Authors:** Francesca Puledda, María Dolores Villar-Martínez, Peter J. Goadsby

**Affiliations:** ^1^Headache Group, Wolfson Centre for Age-Related Diseases (CARD), SLaM Biomedical Research Centre, Institute of Psychiatry, Psychology and Neuroscience, King's College London, National Institute for Health Research (NIHR)-Wellcome Trust King's Clinical Research Facility, King's College Hospital, London, United Kingdom; ^2^Department of Neurology, University of California, Los Angeles, Los Angeles, CA, United States

**Keywords:** visual snow, visual snow syndrome, acute stroke, cerebellum, infarct-vertebral artery dissection

## Abstract

Visual snow syndrome is a novel neurological condition characterized by a panfield visual disturbance associated with several additional symptoms. Although it is usually a continuous and primary disorder, cases of intermittent visual snow have been described in the literature, as well as rare secondary forms. This report is the first description of a case of intermittent visual snow syndrome, which transformed into a persistent form following a posterior circulation stroke due to vertebral artery dissection. At 1 and 2 years after experiencing the acute cerebellar infarct, the patient's only neurological sequalae was visual snow. This case provides a description of how visual snow syndrome may be caused by an underlying brain disorder, and highlights the importance of the cerebellum in the pathophysiology of this relatively unknown condition. It further shows evidence of how existing predispositions might be relevant to the development of visual snow, in certain subjects and following specific circumstances.

## Introduction

Visual snow (VS) is a neurological disorder typically manifest as a panfield visual disturbance consisting of uncountable small dots that are continuously moving. Visual snow syndrome (VSS) manifests as that visual disturbance in association with other symptoms, such as palinopsia (1). VS is reported to occur in about 3.7% of the population (2). VSS is typically persistent after onset, either from as early as a patient can remember, or from a particular day, with only variation by degree over time (3). One case of occipital stroke precipitating a change from intermittent to persistent VS has been reported that had their visual symptoms resolve after 1 year (4). Here we present a case of intermittent VSS that became persistent after a posterior circulation stroke involving the cerebellum.

## Case Description

A 44-year-old male came to our attention in the headache clinic at King's College Hospital, London in October 2020. One year prior, in October 2019, he had had a posterior circulation ischemic stroke, for which the history follows.

### Stroke

One afternoon, about 5–10 min after some physical exercise, he started noticing some gait imbalance, external vertigo and visual disturbance characterized by a right-sided hemianopia and possibly diplopia. These symptoms were self-limited and lasted in total around 10 min; they had not been preceded by any other unusual sensations. After going to bed that evening, he woke up in the middle of the night feeling extremely dizzy and nauseous. He was not able to sit or stand up on his own, and was immediately taken to A&E. He does not recall the ambulance ride, during which he had reduced consciousness and multiple episodes of vomiting.

**Figure 1:**
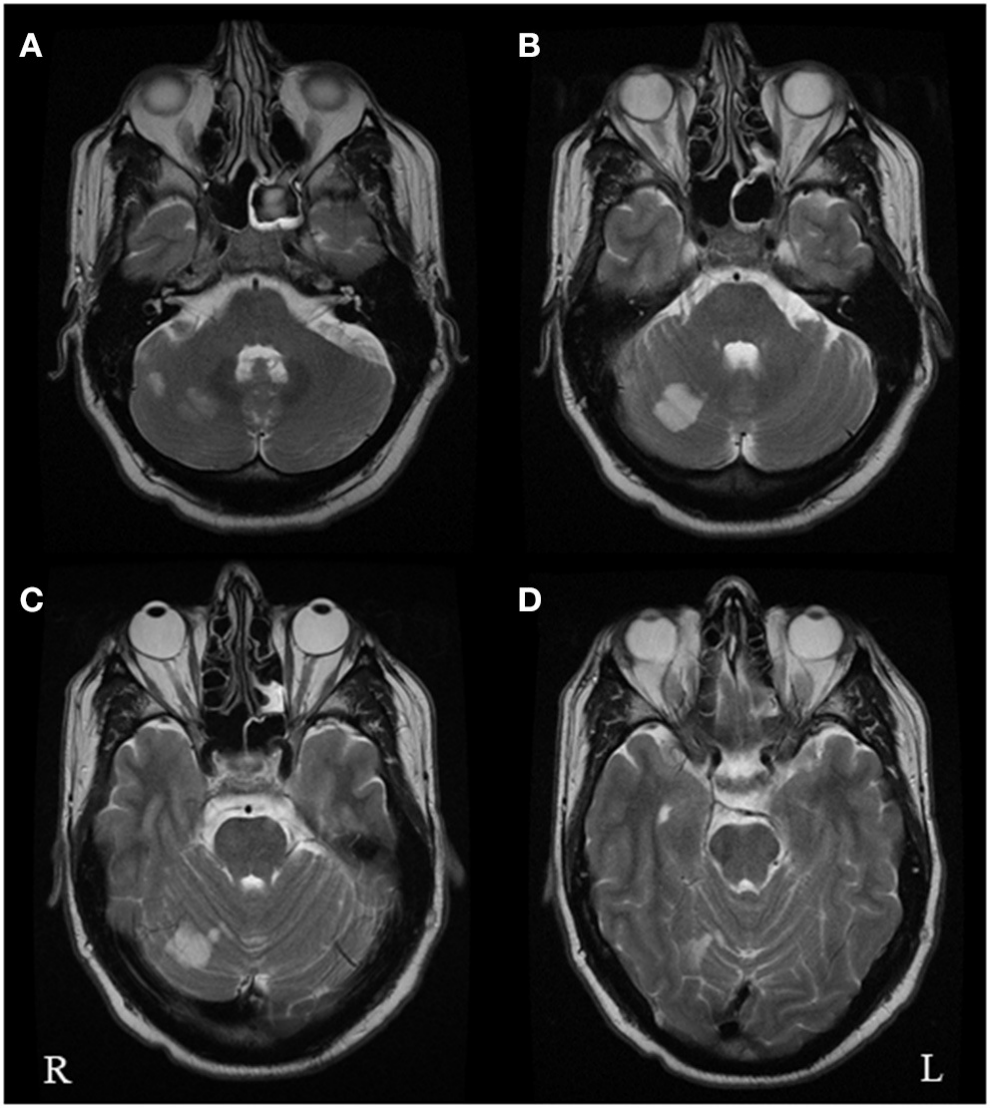
Axial T2 brain MRI images performed in October 2019 following the acute vascular event. The images show multiple foci of restricted diffusion in the right superior cerebellar hemisphere **(A–C)** within the territory of the right superior cerebellar artery, consistent with acute infarcts. There was no restricted diffusion within the left occipital lobe **(D)**.

In hospital he underwent an initial CT scan, which showed an infarct in the right superior cerebellar hemisphere. A further CT angiogram showed evidence of a right vertebral artery dissection. On the following day, an MRI head scan confirmed an acute infarct in the right superior cerebellar artery territory (Figure 1). A repeat CT angiogram 6 months after the acute episode showed that the right vertebral dissection had fully healed.

The patient was initially treated with aspirin followed by clopidogrel while in hospital. However, further screening showed evidence of an atrial arrhythmia in the form of atrial flutter and atrial fibrillation, following which he was started on apixaban. The atrial arrythmia was considered coincidental. The remaining tests he underwent were unremarkable.

### Persistent Visual Symptoms

His present symptoms started perhaps a day after the onset of the stroke, although the patient cannot exclude it being present from the very onset, given his more serious symptoms and the lowered level of consciousness he was experiencing at the time, which might have masked the visual disturbance. In the beginning, and up to 4–5 months after the event, he remembers the visual symptoms being less intrusive and possibly not constant. They have however been quite clearly continuous and unvaried for the 6 months prior to review.

In the headache clinic he reported a continuous unremitting multi-colored and flashing TV-like static, present in the entire visual field. The static was more noticeable when looking at a darker area, and could disappear for a few seconds when he looked at a well-light bright area. In addition, he described prominent afterimages. without trailing, and blue field entoptic phenomenon, floaters, spontaneous photopsia, self-light of the eye, photophobia (particularly when tired) without photic allodynia, and some degree of nyctalopia. He had no tinnitus. He reported the static to be the most bothersome symptom, being quite distracting and made working at a computer screen as part of his job very tiresome. Stress could worsen the static. Prescription sunglasses helped with photophobia but not with the static.

The patient was subsequently reviewed in October 2021, at 2 years from the acute vascular event, and his symptomatology remained completely unchanged.

### Intermittent, Pre-stroke, Visual Symptoms

Before October 2019, the patient reported having the same panfield multi-colored static and associated symptoms, with the only difference that they were not continuous. He remembered first noticing this disturbance in his teenage years, when he would get regular episodes of visual static perhaps once per month, lasting about 12 h and which would usually go away with sleep. He had no headache with these episodes, but he would get photophobia and feel quite tired. He had regular episodes throughout his twenties and thirties, up until October 2019.

### Other History

As general medical history, he had a diagnosis of hypercholesterolemia and gout and occasional migraine without aura, coming perhaps once per year. He would sometimes take paracetamol for the migraine and had never needed a preventive in the past.

He was an ex-smoker up to 10 years prior, of <10 cigarettes a day. He reported drinking about 4–5 units of alcohol per week; he was a heavier drinker up to 4 years prior, with weekly binges. He reported no current use of recreational drugs, including cannabis; he had occasionally used ecstasy in his mid-twenties. Importantly, his episodic visual snow had started at least 10 years prior to any recreational drug use; however, he does recall some visual symptoms such as trailing following previous ecstasy intake. With regards to family history, his father had a previous stroke, while his sister, mother and maternal grandmother all reportedly had migraine.

His ongoing medication at the time he was reviewed in our clinic was apixaban 5 mg BD for atrial fibrillation/secondary stroke prevention, atorvastatin 40 mg OD, allopurinol 200 mg and colchicine 5 mg BD for gout. He had not taken any medication for his visual disturbance since the onset.

## Discussion

This clinical case is unique as it represents the only report in the literature of recurring episodic visual snow, then becoming chronic following a cerebellar stroke. It also offers some very important insight on VSS pathophysiology, particularly highlighting the role of the cerebellum.

Visual snow is a newly defined neurological entity consisting of an unremitting panfield visual disturbance described as numerous tiny flickering dots, or static (5). On the same clinical spectrum of visual snow is VSS (3), in which the static is accompanied by intrusive visual symptoms of the type of palinopsia, enhanced entoptic phenomena, photophobia, and nyctalopia (6). The pathophysiology of visual snow and its associated syndrome are still unclear, and available treatment is lacking (7, 8). Recently, however, neuroimaging, neurobehavioral and electrophysiological studies have helped to define what is most likely a complex network disorder characterized by a disturbance in the interaction between different areas of the visual system, as well as other brain regions involved in visual and sensory processing (9–14).

There has only been one report of ischemic stroke associated with visual snow phenomenon in the literature, reported by Catarci (4). Similar to our case, in this description of a 74 year old patient with occipital infarct following a right posterior cerebral artery occlusion, VS symptoms changed from transient to continuous (although occupying only one part of the visual field) immediately after the acute vascular event. Interestingly, one case with an opposite outcome has also been described, where a haemorrhagic stroke of the left thalamus was followed by a 1-week resolution of visual snow symptoms, in a 25 year old female (15).

**Figure 2:**
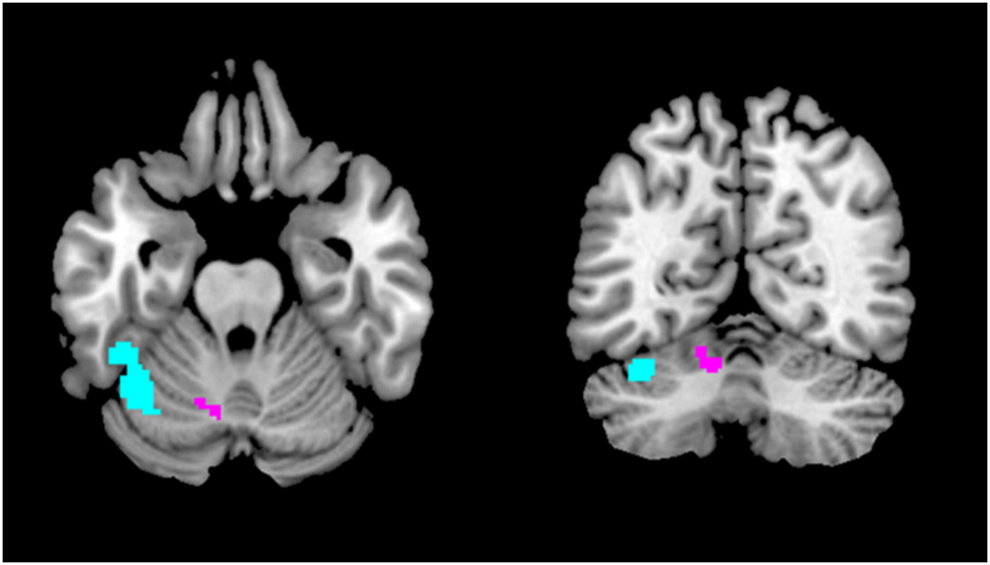
Regions of the cerebellum implicated in VSS pathophysiology. The area in purple showed gray matter volume increase with VBM (16), whereas the region in light blue showed increased regional cerebral blood flow with ASL (17) and altered functional connectivity to the posterior cingulate cortex with resting state fMRI (18).

The region of the infarct in our patient corresponded to the territory of the superior cerebellar artery, and in anatomical terms to cerebellar Crus I-lobule VI. Importantly, very similar regions have recently been implicated in visual snow pathophysiology. A structural MRI study by our team found a gray matter volume increase specifically in Crus I-lobule VI (16) (Figure 2). Functional MRI has also demonstrated cerebellar changes in VSS: arterial spin labeling (17) detected increased perfusion in the lateral and posterior cerebellum (Figure 2); whereas a further functional connectivity analysis (18) revealed that this same cerebellar region, which is known to form part of the dorsal attentional network (19), showed altered connectivity to the posterior elements of the default mode network. These functional changes, which implicate abnormal activity within networks that regulate major brain functions, were found regardless of the underlying brain state, signifying that they might have a relevant role in the basic neurobiology of VSS.

Due to its extensive circuitry with cortical and sub-cortical structures of the prefrontal and parietal regions, the posterior cerebellum plays a key role not only in motor functions but also in sensori-motor and cognitive integration (20–22). The Crus I-lobule VI regions in particular have been described as part of the “cognitive cerebellum” (23), which can regulate complex functions such as attention, decision making, visual working memory and even emotional processing (24). Cerebellar dysfunction has been implicated in several pathological brain states, including depression (25) and autism (26). It is thus not entirely surprising that a similar network-type disorder such as visual snow syndrome (27) might be caused by at least a partial involvement of this complex brain region.

Although much still needs to be understood about visual snow, it is becoming clear that some forms of the condition might arise in the aftermath of a specific incident, such as a changes in headache comorbidity (1), an infection (28) or the start of new medication (29). In these cases, it is possible to hypothesize that some underlying vulnerability might exist in specific subjects, allowing an inciting event to easily trigger the phenomenon (15). The presence of episodic visual snow in our patient, previously dismissed as a somewhat normal perception, seems to characterize him as one of these predisposed individuals; it then took an independent brain insult in a region directly involved in visual snow pathogenesis to facilitate the resurfacing of the dysfunction, causing a complete and continuous manifestation of the disorder.

## Data Availability Statement

The original contributions presented in the study are included in the article/supplementary material, further inquiries can be directed to the corresponding author/s.

## Author Contributions

FP wrote the first draft of the manuscript. MDVM and PG edited the final version of the manuscript. All authors reviewed and discussed the case, contributed to the article, and approved the submitted version.

## Funding

This work was funded by NIHR SLaM Biomedical Research Centre at South London, Maudsley NHS Foundation Trust, King's College London (IS-BRC-1215-20018), and the Visual Snow Initiative. FP is funded by the King's Prize Fellowship and Anthony Mellows medal.

## Conflict of Interest

The authors declare that the research was conducted in the absence of any commercial or financial relationships that could be construed as a potential conflict of interest.

## Publisher's Note

All claims expressed in this article are solely those of the authors and do not necessarily represent those of their affiliated organizations, or those of the publisher, the editors and the reviewers. Any product that may be evaluated in this article, or claim that may be made by its manufacturer, is not guaranteed or endorsed by the publisher.
